# Sexual dimorphism in immune response genes as a function of puberty

**DOI:** 10.1186/1471-2172-7-2

**Published:** 2006-02-22

**Authors:** Rebecca Lamason, Po Zhao, Rashmi Rawat, Adrian Davis, John C Hall, Jae Jin Chae, Rajeev Agarwal, Phillip Cohen, Antony Rosen, Eric P Hoffman, Kanneboyina Nagaraju

**Affiliations:** 1 Division of Rheumatology, Department of Medicine, Johns Hopkins University School of Medicine, Baltimore, MD, USA; 2 Research Center for Genetic Medicine, Children's National Medical Center, 111 Michigan Ave, NW, Washington DC, 20010, USA; 3 National Institutes of Health, Bethesda, MD, USA; 4 University of Pennsylvania, Philadelphia, PA, USA

## Abstract

**Background:**

Autoimmune diseases are more prevalent in females than in males, whereas males have higher mortality associated with infectious diseases. To increase our understanding of this sexual dimorphism in the immune system, we sought to identify and characterize inherent differences in immune response programs in the spleens of male and female mice before, during and after puberty.

**Results:**

After the onset of puberty, female mice showed a higher expression of adaptive immune response genes, while males had a higher expression of innate immune genes. This result suggested a requirement for sex hormones. Using *in vivo *and *in vitro *assays in normal and mutant mouse strains, we found that reverse signaling through FasL was directly influenced by estrogen, with downstream consequences of increased CD8^+ ^T cell-derived B cell help (via cytokines) and enhanced immunoglobulin production.

**Conclusion:**

These results demonstrate that sexual dimorphism in innate and adaptive immune genes is dependent on puberty. This study also revealed that estrogen influences immunoglobulin levels in post-pubertal female mice via the Fas-FasL pathway.

## Background

The incidence and severity of human diseases vary between the sexes: For example, autoimmune diseases are generally more common in females than in males and are most marked in women of childbearing age [1-3]. Thus, it appears that susceptibility to autoimmunity is expressed at the time of puberty. Puberty is a period of intense molecular, physiological and anatomical reorganization in the body, and the hormonal changes occurring at the time of puberty lay the framework for biological differences that persist throughout life and may contribute to the variable onset and progression of disease in males and females [4]. Sex-related differences in disease susceptibility have also been observed in several mouse models of infectious and autoimmune diseases and may be related to differences in the expression patterns of immune response genes [5,6].

Immune responses are sexually dimorphic, both in type and magnitude. Two general systems of immunity to infectious agents have been selected during evolution: innate (natural) immunity, and acquired (adaptive or specific) immunity. The innate immune system uses proteins encoded in the germline (on macrophages, mast cells, natural killer cells) to recognize conserved products of infectious non-self (i.e., microbial pathogens), but not non-infectious self (i.e., host proteins) [7,8]. In contrast to this relatively inflexible system is the almost infinitely adaptable immune system of lymphocytes [9]. These two systems are known to interact closely with each other: For example, cellular and soluble components of innate immunity help the adaptive immune response to select and respond to appropriate antigens. Even though these two systems are very well studied, there is a paucity of literature on gender differences as a function of age. Understanding the basis of sex differences in immune response genes is important for developing new approaches to prevention, diagnosis and treatment of infectious and autoimmune diseases.

We studied sexual dimorphism in immune response genes in C57Bl/6 (B6) mice because B6 mice do not spontaneously develop autoimmune diseases. However, when autoimmune-susceptible loci are transferred onto a B6 background, the mice readily manifest a disease phenotype, including profound sex differences in disease severity [10,11]. We have now investigated the sex differences in immune response genes in the spleens of pre-pubertal, pubertal and post-pubertal male and female B6 mice using global gene expression profiling. Our data indicate that there is a clear sexual dimorphism after puberty in innate and adaptive immune genes. We have also identified one such pathway, reverse signaling through FasL, as a possible source of the sexual dimorphism in immunoglobulin (Ig) levels that is seen between males and females, since this pathway is affected by estrogen levels.

## Results

### Gene expression in spleen during puberty

To define puberty-related changes in immune system function, we performed a series of gene expression profiling experiments using 12,000 gene highly redundant oligonucleotide arrays (Affymetrix U74Av2) on spleens of normal pre-pubertal (3- to 4-week-old), pubertal (6- to 9-week-old) and post-pubertal (24- to 28-week-old) female and male C57BL/6 (B6) mice to identify gender- and age-specific expression programs. We used a microarray data analysis approach that was optimized for signal/noise in tissue samples [12]; unsupervised hierarchical clustering analyses of these samples showed that the biological variables (age, sex) were dominant over technical and inter-individual variables [12,13]. Genes involved in cell signaling, cell growth, cell differentiation, extracellular matrix synthesis, morphogenesis, vesicle trafficking, oncogenesis and immune responses were up-regulated during puberty in both male and female spleens (e.g., septin family genes, GDNF, R-ras, Ets family transcription factors, Rab family GTPases, alpha catenin, TGF beta, prolactin-like protein, tenascin-X and IKaros) (see Additional file 1). Other genes specifically down-regulated during puberty belonged to the p53 tumor suppressor pathway, chromatin remodeling, cell cycle, DNA repair, replication and transcription categories (e.g., RAD23a, Dnmt1, Ki 67, mBlm, cdc6 and sak) (see Additional file 2). The majority of puberty-driven gene programs in both female and male spleens were involved in erythropoiesis (e.g., erythropoietin receptor, Duffy blood group), consonant with the fact that erythropoiesis is exceptionally active during puberty [14]. These results suggest that puberty, which enables the initiation and development of female and male reproductive capabilities, is a period of intense molecular reorganization in the spleen, probably in response to gender-specific hormones.

### Post-pubertal sex differences in immune response genes

**Figure 1:**
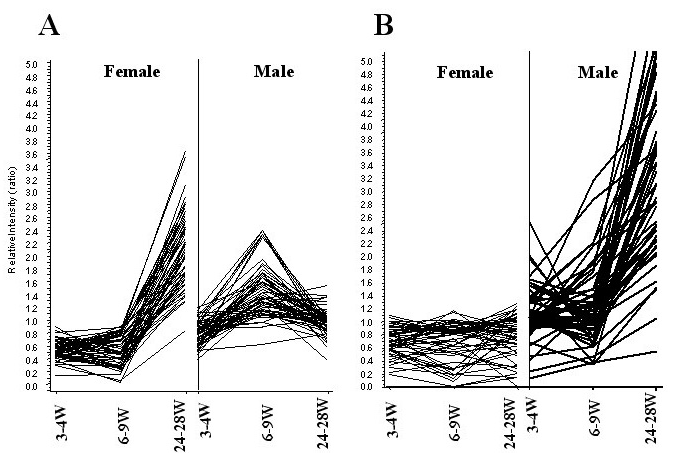
**Post-pubertal, sexually dimorphic gene expression patterns in spleen**. Shown is a data visualization of gender-specific gene expression patterns in normal mouse spleen. Six C57BL/6 mouse spleens from each sex at the pre-pubertal (3–4 weeks), pubertal (6–9 weeks) and post-pubertal (24–28 weeks) stages (a total of 36 mice) were analyzed using high-density oligonucleotide arrays. A hierarchical clustering algorithm was applied to group genes based on the similarities in gene expression patterns. The X-axis represents time points (weeks) in female (left) and male (right) mice. The Y-axis represents relative intensity ratios. A: Gene cluster up-regulated in post-pubertal female mice, B: Gene cluster up-regulated in post-pubertal male mice. These clusters were used for subsequent identification of sexually dimorphic immune function pathways.

We then mined the data for explanations for the sexually dimorphic immune response by first clustering for female- and male-specific post-pubertal expression differences (Figure 1), then querying these groups for immune-related mRNAs. Assignment of functions to cluster members suggested that post-pubertal male mice preferentially expressed genes involved in innate immunity (see Table 1 and Additional file 3 for full list). The expression of these innate immunity genes was significantly decreased in post-pubertal female mice. These results suggest that considerable remodeling of splenic immune function occurs at the time of puberty, with the result that sexual dimorphism in immunity is permanently established in post-pubertal life, with adult males showing a predominantly primitive immune (innate) response and adult females being relatively deficient in these responses. In contrast, post-pubertal female mice preferentially expressed adaptive immune response genes (Table 2, see Additional file 3), and expression of the same genes occurred at lower levels in post-pubertal male mice.

**Table 1:** Innate immune response genes differentially expressed in post-pubertal B6 male mice.

**Acc#**	**Fold Change**	**Chr**	Gene Name	Function
X03505	95.9	7	Serum Amyloid A	Anti-inflammatory
X70057	12.9	14	Cathepsin G	Phagocytosis
X15313	4.4	11	Myeloperoxidase	Phagocytosis
M94584	6.7	3	Chitinase 3-like 3	Monocyte maturation
X81627	3.8	2	24p3	Immunomodulation
U04962	5.4	10	Neutrophil elastase2	Phagocytosis
U43525	6.7	10	Proteinase 3	Phagocytosis
J03298	1.7	9	Lactotransferrin	Anti-inflammatory
AA144469	4.1	7	Interferon-inducible protein 1-8p	Immunomodulation
AF099977	2.3	11	mSLFN4 (schlafen 4)	Thymocyte maturation
M27008	10.3	4	Alpha-1 acid glycoprotein	Anti-inflammatory
X70920	2.7	15	Granulocyte maturation Ly-6G.1	Phagocytosis
X59769	3.4	1	IL-1r2	Anti-inflammatory
M69260	3.5	19	Lipocortin 1 (Annexin A1)	Anti-inflammatory
X78545	1.9	14	Mast cell protease 8	Mast cell function
M96827	3.6	8	Haptoglobin	Anti-inflammatory
AF076482	3.6	7	Peptidoglycan recognition protein	Phagocytosis
AF071180	3.0	17	Formyl peptide receptor-1 like receptor	Neutrophil migration
AF051367	1.7	16	Integrin beta subunit-like cell-surface protein	Anti-inflammatory
U49513	4.8	11	Mip 1 gamma (CCl 9)	Neutrophil migration
U29678	5.7	9	CCR-1	Chemotaxis of monocytes
AA596710	4.8	4	Leukotriene B4 12-hydroxydehydrogenase	Anti-inflammatory
J05018	6.6	1	High affinity IgE receptor alpha subunit	Mast cell activation
X16490	2.7	1	Plasminogen activator inhibitor 2 (Serpin b2)	Anti-inflammatory
U05265	3.2	10	gp49B	NK and mast cell function
X66449	2.1	3	Calcyclin	Monocyte differentiation
X15592	2.5	13	Ctla-2-beta	Mast cell function

**Table 2:** Adaptive immune response genes differentially expressed in post-pubertal B6 female mice.

**Acc#**	**Fold Change**	**Chr**	**Gene name**	**Function**
AE000665	2.4	6	TCR beta locus	T cell signaling
X00651	4.0	6	Ig-kappa light chain V-J kappa region	Immunoglobulin
U19315	4.2	6	Immunoglobulin kappa light chain region	Immunoglobulin
M15593	4.9	6	Ig kappa chain 7B6 mRNA	Immunoglobulin
AF037206	1.7	3	RING zinc finger protein	Lymphocyte development
M86751	2.1	6	Ig L-chain gene variable region	Immunoglobulin
X94420	4.7	12	IgA V-D-J-heavy chain	Immunoglobulin
V00793	141.8	12	IgG1	Immunoglobulin
X67210	13.3	12	IgG2	Immunoglobulin
L43568	5.3	6	B-cell receptor gene	B cell signaling
U37386	3.0	2	Carboxyl ester lipase	Lymphocyte maturation
M55412	1.6	19	Guanine nucleotide BP, alpha polypeptide	Lymphocyte signaling

We found no differences in the expression of adaptive immune response genes in pre-pubertal male and female mice (Figure 1A). Even though the basal levels of some innate immune response pathway genes were higher in pre-pubertal male than pre-pubertal female mice, these differences were not statistically significant (Figure 1B). The difference in the expression of these genes cannot be explained by their chromosomal localization, because these genes are predominantly encoded by autosomes (Tables 1 and 2). However the post-pubertal nature of these differences clearly suggests that indeed many of these genes are influenced by sex hormones.

**Figure 2:**
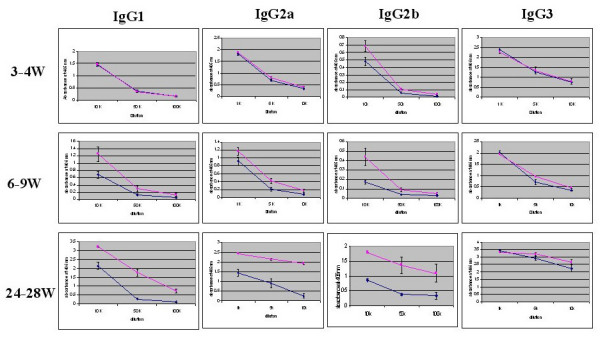
**Serum immunoglobulin levels in pre-pubertal, pubertal and post-pubertal female and male C57BL/6 mice**. Serum samples from 6–8 C57BL/6 mice at 3–4 weeks, 6–9 weeks and 24–28 weeks were collected and assayed by ELISA. The samples were tested at dilutions of 1:5,000; 1:10,000; 1:50,000 and 1:100,000. The line graph presents the mean absorbance at 405 nm ± SE for males (blue line) and females (pink line).

**Figure 3:**
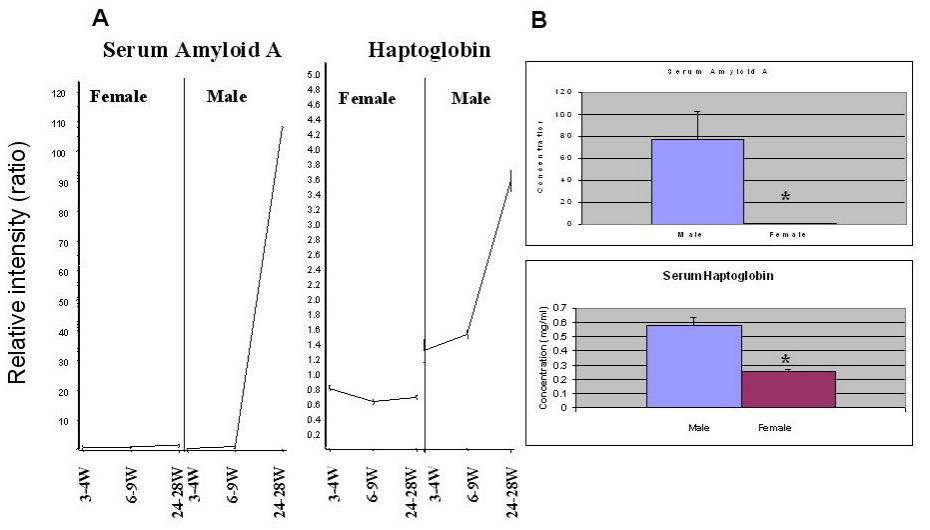
**Acute phase proteins in post-pubertal mice**. A: Serum amyloid A and haptoglobin mRNA expression patterns in pre-pubertal, pubertal and post-pubertal mice. The X-axis represents time points (weeks) in female and male mice, and the Y-axis represents relative intensity ratios. B: Serum amyloid A and haptoglobin protein levels in post-pubertal C57BL/6 mice. Serum amyloid A levels in 24- to 28-week-old C57BL/6 mice. Serum samples (1:200) along with known standards were assayed in 96-well ELISA plates. The concentrations of the test samples were determined from the standard curve by multiplying the interpolated values by the dilution factor (n = 10/sex: male vs. female [μg/ml mean ± SE]: 77.48 ± 25.26 vs. 0.38 ± 0.12; p = 0.009). The working assay range was 11.8-190 μg/ml. Normal serum levels of SAA are generally less than 20 μg/ml. Serum haptoglobin levels were assayed using a colorimetric assay. Normal murine haptoglobin levels range from 0–0.1 mg/ml, and these increase in the acute phase to 0.3–2.0 mg/ml. The assay has a sensitivity of 0.05 mg/ml haptoglobin. (n = 10/sex: male vs. female [mg/ml mean ± SE]: 0.578 ± 0.059 vs. 0.254 ± 0.013; p = 0.00004).

We then confirmed the sexual dimorphism of adaptive (immunoglobulin isotypes) and innate (serum amyloid A and haptoglobin) immune response proteins in male and female B6 mice. Serum Ig levels (IgG1, IgG2a, IgG2b, and IgG3) showed no significant differences between pre-pubertal male and female mice, but differences began to appear at puberty and became significant in post-pubertal mice (Figure 2). These differences were particularly striking for IgG1, IgG2a and IgG2b isotypes. Significantly higher serum IgM, kappa light chain, but not lambda light chain expression was observed in post-pubertal female mice (data not shown). No differences in IgG3 or IgA were observed between male and female mice in any age group. We also examined two innate immune response proteins, serum amyloid A and haptoglobin, both of which showed increased levels in post-pubertal male mice as compared to age-matched female mice (Figure 3).

Because of the striking differences in several innate immune response genes in post-pubertal mice (Figure 1B, Table 1) we hypothesized that male and female mice should respond differently to innate immune stimuli. We used well-characterized innate immune ligands (i.e., lipopolysaccharide [LPS; TLR 4], lipoteichoic acid [LTA, TLR 2], poly I:C [TLR 3], Imiquimod [TLR 7/8], Pam_3_CSK_4 _[TLR 1], and T1 CpG DNA [TLR 9]) to stimulate splenocytes of post-pubertal mice, then measured their ability to produce various cytokines and chemokines that are known to affect the innate and adaptive immune systems, including IL-1α, IL-1β, IL-6, IL-10, IL-12, IL-18, MCP-1 (JE) and RANTES [15,16]. We found that the chemokine, MCP-1, is constitutively increased in female mice without any treatment (male vs. female (pg/ml); 3.7 ± 0.4 vs. 17.3 ± 5.8; p < 0.05) and these levels were further elevated after treatment with Imiquimod (male vs. female (pg/ml); 28.6 ± 4.5 vs. 65.6 ± 13.9; p < 0.05) and T1CpG DNA (male vs. female (pg/ml); 9.6 ± 3.1 vs. 43.5 ± 12.1; p < 0.05). We also found that IL-6 and IL-10, cytokines that influence the adaptive arm (i.e., antibody production), were significantly increased in female mice after treatment with T1CpG DNA (IL-6: male vs. female (pg/ml); 169.5 ± 21 vs. 279.2 ± 44.7; p < 0.05 and IL-10: (male vs. female (pg/ml); 51.5 ± 9.8 vs. 80.6 ± 10.5; p < 0.05). In contrast, IL-1α and IL-1β, cytokines affecting the innate immune system, were significantly increased in males after LTA treatment (IL-1α : male vs. female (pg/ml); 21.9 ± 0.9 vs. 14.6 ± 2.2; p < 0.05 and IL-1β 25.3 ± 4.3 vs. 11.9 ± 2.7; p < 0.05). Treatment with either Pam_3_CSK_4_, LPS or poly I:C did not lead to a significant sexual dimorphism in the cytokine/chemokine profiles that were examined (Table 3).

**Table 3:** TLR ligand-induced cytokine/chemokine production in post-pubertal mice shows sexual dimorphism.

**TLR ligand**	**Cytokine/Chemokine**	**Male (pg/ml)**	**Female (pg/ml)**
Media	IL-1α	2.6 ± 0.5	3.8 ± 0.8
	IL-1β	7.2 ± 2.3	6.5 ± 1.3
	IL-6	39.7 ± 4.1	39.4 ± 10.2
	IL-10	20.2 ± 3.5	27 ± 2.8
	IL-12	4.1 ± 1.4	4.7 ± 1.1
	IL-18	80.1 ± 17.2	46 ± 11.2
	MCP-1	3.7 ± 0.4	17.3 ± 5.8*
	RANTES	159.2 ± 56.5	171.4 ± 54.2
LPS	IL-1α	24.5 ± 1.8	24.8 ± 5.2
	IL-1β	29.1 ± 4.1	23.8 ± 3.6
	IL-6	848.7 ± 154.4	1132.7 ± 323
	IL-10	1484.5 ± 56.2	1960.5 ± 316.4
	IL-12	12.7 ± 1.0	13.3 ± 1.1
	IL-18	150.8 ± 10.8	151.8 ± 23.9
	MCP-1	14.8 ± 2.7	21.2 ± 4.4
	RANTES	937.5 ± 369.8	1202.3 ± 567.4
LTA	IL-1α	21.9 ± 0.9*	14.6 ± 2.2
	IL-1β	25.3 ± 4.3*	11.9 ± 2.7
	IL-6	214.2 ± 36.1	174.2 ± 48.3
	IL-10	199.1 ± 21.3	210.7 ± 43.8
	IL-12	9.5 ± 1.3	7.4 ± 0.5
	IL-18	118.3 ± 6.0	94.1 ± 16.7
	MCP-1	26.5 ± 3	32.2 ± 7.5
	RANTES	675.0 ± 228.5	772.4 ± 323.6
Poly I:C	IL-1α	24.2 ± 1.9	20.0 ± 3.0
	IL-1β	20.7 ± 2.1	13.8 ± 3.7
	IL-6	201.4 ± 36.7	192.2 ± 45.59
	IL-10	257.6 ± 17.8	298.1 ± 37.8
	IL-12	6.7 ± 0.5	7.5 ± 0.5
	IL-18	92.6 ± 12.2	105.8 ± 18.0
	MCP-1	24.6 ± 2.3	31.3 ± 7.1
	RANTES	644.4 ± 236.3	980.7 ± 362.3
Imiquimod	IL-1α	5.9 ± 0.8	7.3 ± 0.7
	IL-1β	29.4 ± 7.2	28.2 ± 1.5
	IL-6	4831.3 ± 1210.29	5256.1 ± 881.4
	IL-10	1232.3 ± 272.3	1578.7 ± 145.9
	IL-12	12.9 ± 1.7	14.5 ± 1.6
	IL-18	137.1 ± 19.8	136.3 ± 15.0
	MCP-1	28.6 ± 4.5	65.6 ± 13.9*
	RANTES	632.1 ± 292.6	679.2 ± 273.1
Pam_3_CSK_4_	IL-1α	10.3 ± 6.9	3.2 ± 0.4
	IL-1β	12.5 ± 3.5	6.5 ± 0.2
	IL-6	272.9 ± 29.6	262.6 ± 59.4
	IL-10	295.3 ± 30.6	351.8 ± 38.5
	IL-12	5.4 ± 1.1	6.6 ± 1.2
	IL-18	47.8 ± 17.0	80.9 ± 14.8
	MCP-1	15.7 ± 1.4	40.2 ± 12.5
	RANTES	195.7 ± 68.3	279.2 ± 94.0
T1 CpG	IL-1α	3.5 ± 1.0	3.5 ± 0.7
	IL-1β	9.5 ± 2.2	7.7 ± 1.5
	IL-6	169.5 ± 21	279.2 ± 44.7*
	IL-10	51.5 ± 9.8	80.6 ± 10.5*
	IL-12	6.0 ± 0.6	7.2 ± 1.2
	IL-18	58.4 ± 7.1	96.5 ± 18.0
	MCP-1	9.6 ± 3.1	43.5 ± 12.1*
	RANTES	403.4 ± 158.7	619.3 ± 218.3

*Indicates statistical signifigance (p < 0.05)

### Role of Fas/FasL pathway in generating sexual dimorphism in Ig gene expression

**Figure 4:**
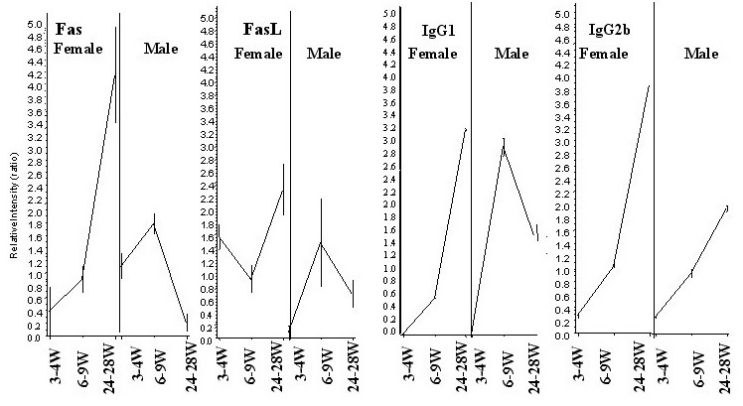
**Immunoglobulin isotypes, Fas, and FasL genes show similar expression patterns**. Nucleating hierarchical clustering with IgG1 and IgG2b revealed Fas and FasL as co-regulated transcripts in post-pubertal female mouse spleens. These data drove the hypothesis that a non-apoptotic role of Fas/FasL might be responsible for downstream differential IgG isotype expression. The X-axis represents time points (weeks) in female and male mice, and the Y-axis represents relative intensity ratios.

Since functional gene clusters that participate in the same biological pathway share "spatial" and "temporal" expression profiles, we looked for transcripts that shared a temporal expression pattern with the Ig genes. We found that the Fas and FasL pathway genes were regulated in their expression pattern in a manner similar to that observed for the IgG isotypes (Figure 4). Therefore, we explored the possibility that the Fas-FasL pathway may be associated in generating differences in immunoglobulin levels in post-pubertal male and female mice.

**Figure 5:**
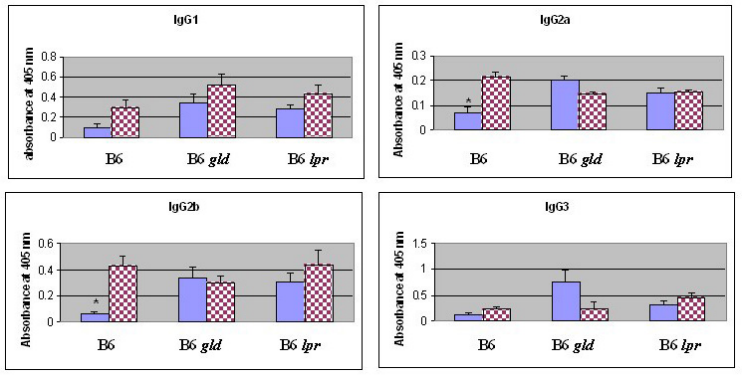
**Differences in serum immunoglobulin IgG2a and IgG2b isotypes are abolished in post-pubertal B6 *lpr *(Fas-deficient) and B6 *gld *(FasL-deficient) mice**. Serum samples from 16- to 20-week-old male and female (5–6 mice/sex) B6, B6 *lpr *and B6 *gld *mice were assayed by ELISA. The samples were diluted at 1:5,000 (IgG2a); 1:10,000 (IgG3); 1:50,000 (IgG1 and IGg2b). Bar graphs represent mean absorbance at 405 nm ± SE. Significant differences for IgG2a (p = 0.0009) and IgG2b (p = 0.00005) between post-pubertal male (blue bar) and female (checker red bar) mice are shown with asterisks.

To investigate the role of this pathway *in vivo*, we estimated the levels of Ig isotypes in post-pubertal male and female mice that were defective in Fas (*lpr*, insertion of an early transposable element into the second intron) or FasL (*gld*, point mutation in the C-terminal region) on a B6 background [17,18]. The differences in IgG2a and IgG2b levels in post-pubertal male and female B6 mice were abolished in both Fas-defective (B6 *lpr*) and FasL-defective (B6 *gld*) mice, strongly suggesting that the Fas-FasL pathway is involved in generating the differences in immunoglobulin levels seen in post-pubertal male and female mice (Figure 5).

Because Fas/FasL expression was more pronounced in post-pubertal female mice than in males (Figure 4), we hypothesized that these genes may be influenced by female sex hormones. A putative estrogen response element has been previously described in the FasL promoter, and FasL exists mainly in a membrane-bound form on activated CD8^+ ^cells of the T cell lineage [19]. Thus, we measured FasL by flow cytometry and found that its expression was enhanced by a physiological dose of estrogen (10^-8^M) on these activated CD8^+ ^T cells (see Additional file 4). Previous studies had shown that reverse signaling through FasL leads to increased proliferation of CD8^+ ^but not CD4^+ ^T cells [20,21]. We therefore considered the possibility that increased estrogen levels during post-pubertal life enhance FasL expression and lead to downstream activation of CD8^+ ^T cells. These activated CD8^+ ^T cells would be expected to secrete growth factors and cytokines that, in turn, would enhance immunoglobulin gene expression in these post-pubertal female mice.

### Effect of activated CD8^+ ^T cell culture supernatants on IgG isotype expression

**Figure 6:**
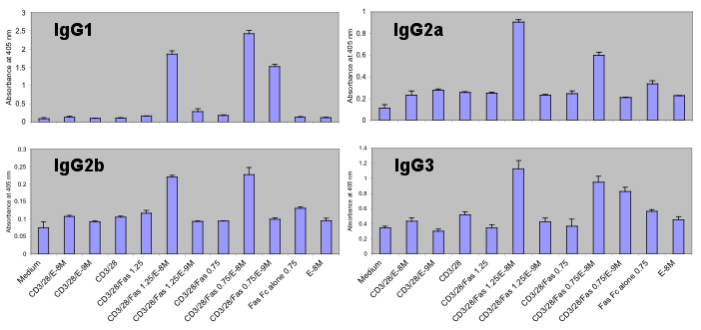
**Effect of activated CD8^+ ^T cell supernatants on Ig expression *in vitro *antibody synthesis**. A representative example of 3 separate experiments is shown. T cells (5 × 10^5^) were stimulated for 18 h with various stimulants either individually or in combination (CD3/CD28, anti-CD3 and anti-CD28; E, estrogen, (10-8 M and 10-9 M), Fas-Fc (μg/ml: 0.75 and 1.25). The culture supernatants from activated CD8^+ ^T cells were collected and added (200 μl) to 1.5 × 10^6 ^splenocytes (800 μl) and incubated for 10 days. The culture supernatants were assayed for various immunoglobulin isotypes using ELISA. Graphs represent mean absorbance of triplicates at 405 nm ± SD.

To investigate and further confirm this hypothesis, we purified CD8^+ ^T cells from post-pubertal female mice and activated them with plate-bound Fas Fc, along with anti-CD3/CD28, in the presence and absence of estrogen for 18 h. Culture supernatants from the activated CD8^+ ^T cells were collected and incubated with splenocytes in an *in vitro *immunoglobulin synthesis assay, and IgG isotypes were estimated on day 10 using an indirect ELISA. Supernatants from Fas-Fc/CD3/CD28-activated CD8^+ ^T cells cultured in the presence of estrogen showed enhanced expression of IgG isotypes (IgG1, IgG2a, IgG2b and IgG3). These results demonstrate that estrogen increases the reverse signaling through FasL in CD8^+ ^T cells, leading to the secretion of growth factors that support increased IgG isotype expression (Figure 6). The effects of estrogen were also shown to be Fas- FasL pathway dependent, since the addition of estrogen alone produced no differences in IgG expression. Therefore, it is expected that this type of signaling represents one mechanism by which sexual dimorphism is expressed at the molecular level.

**Figure 7:**
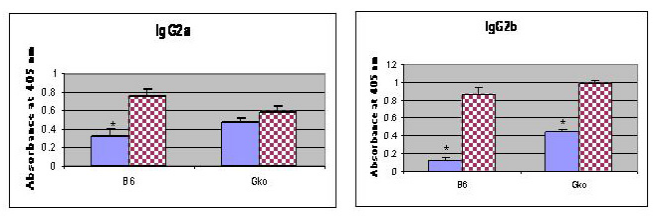
**Differences in serum IgG2a but not IgG2b levels are abolished in B6 IFN-gamma knockout mice (B6 GKO)**. Serum samples from 16- to 20-week-old male and female (4–6 mice/sex) B6 and B6 GKO mice were assayed by ELISA. Graphs represent mean absorbance at 405 nm ± SE. Significant differences for B6 IgG2a (p = 0.005), B6 IgG2b (p = 0.003) and B6 GKO IgG2b (0.001) between post-pubertal male (blue bar) and female (checker red bar) mice are shown with asterisks.

Since IFN-γ is known to influence IgG2a isotype switching, we also analyzed the CD8^+ ^T cell supernatants for cytokine production and found that IFN-γ was produced at higher levels in the presence of estrogen (CD3/CD28/Fas Fc vs. CD3/CD28/Fas Fc/estrogen (pg/ml): 44169 ± 909 vs. 50318 ± 505, p < 0.01) (see Additional file 5). To define the role of IFN-γ, we evaluated the Ig isotype levels in post-pubertal B6 IFN-γ knockout (GKO) mice and found that the gender differences in IgG2a, but not IgG2b, levels were abolished in these mice (Figure 7). These results indicate that CD8^+ ^T cell-derived cytokines such as IFN-γ are involved in generating the observed differences in Ig levels in post-pubertal mice.

## Discussion

We have shown that male and female mice differ significantly with respect to their immune response genes in post-pubertal life. The innate immune response genes are highly up-regulated in post-pubertal male but not female mice. Post-pubertal male mice also produce higher levels of IL-1α and IL-1β in response to the TLR-2 ligand (Table 3). The biological relevance of these findings can be seen in both infectious and autoimmune disease conditions. Although males are more susceptible than females to many parasitic infections, there are some parasites for which males are more resistant than females and differences in innate and adaptive arms of the immune system may explain this sex reversal. For example, the innate immune response plays a critical role in offering males protection against Toxoplasma *gondii *infection [22,23]. Our data are consistent with the relative deficiency of innate immune response genes in female mice, as evidenced by their enhanced susceptibility to and higher mortality associated with certain parasitic infections (e.g., T. *gondii*). Thus, the relative resistance of the males to T. *gondii *infection is likely explained by their high levels of innate immunity-related proteins. Furthermore, it is also known that the 5-lipoxygenase pathway and leukotrienes are integral components of innate immune cells such as macrophages, mast cells and eosinophils [24]. Recent experiments have clearly demonstrated that 5-lipoxygenase-deficient male mice on an MRL *lpr*/*lpr *background show a marked decrease in survival, further supporting a protective role for innate immune response genes in autoimmune diseases [25].

In contrast, adaptive immune response genes are highly up-regulated in post-pubertal female mice. These mice also produce significantly higher levels of cytokines and chemokine that influence antibody production than do post-pubertal males (Table 3). These findings are particularly relevant to autoimmune diseases, in which the adaptive immune system attacks normal self tissue. We propose that enhanced susceptibility to autoimmune disease in post-pubertal life is the result of an altered ratio of adaptive and innate immune response genes. This hypothesis is in fact supported by the finding that genetic defects in innate immune response genes (complement C1q and serum amyloid P) in mice result in spontaneous autoimmune disease [26-28]. It is known that females produce higher levels of Igs than do male mice in response to a variety of antigens, and these effects have been attributed to sex steroids [29-31]. Our results confirm these findings and further indicate that even non-immunized female mice show significantly elevated levels of various Ig isotype genes, and that the levels are even more enhanced in post-pubertal life.

Fas and FasL genes showed spatial and temporal expression patterns similar to those of immunoglobulin genes. The preferential expression of Fas and FasL in post-pubertal females suggested a role for this pathway in generating sexual dimorphism in immunoglobulin gene expression. The observed post-pubertal sex differences in Ig levels in B6 mice were abolished in B6 *lpr *and B6 *gld *mice, indicating that the post-pubertal levels of specific Ig isotypes are regulated through Fas/FasL pathway.

Genetic defects in both Fas and FasL are known to cause severe lupus like autoimmune disease on the MRL/Mp genetic background. The gender differences in disease severity (mortality, pancreatitis and autoantibodies) in MRL/Mp mice are abolished when Fas (*lpr*) mutation is transferred onto this background, suggesting that MRL *lpr *mice are gender-neutral [32,33]. It is important to note that in a previous study, transferring the C1q deficiency onto the MRL background did not abolish the gender differences [34]. Thus, the defects in the Fas-FasL signaling pathway alone abolish the gender differences in lupus-like autoimmune disease in MRL mice. Further supporting this observation is the finding that *lpr *mice show spontaneous polyclonal B cell activation and lymphadenopathy [35]. The male *lpr *mice showed significant increases in Ig levels, similar to those seen in females (Figure 5). These results are interesting, especially when correlated with the disease-prone MRL *lpr *mouse model of lupus, in which male mice die as early as female mice (50% mortality in both male and female mice by 5.5 months of age). This finding suggests that increased IgG levels in males lead to increases in immune complex-mediated disease, similar to those in female mice.

This hypothesis is further supported by another model of autoimmunity: MRL-Fas ^lprcg ^mice have a phenotype similar to that of MRL *lpr *mice because of a defect in Fas-mediated apoptotic signaling (a single amino acid mutation in the cytoplasmic death domain) [36]. The reverse signaling pathway through FasL is functional because of the intact extracellular domain that interacts with FasL. In fact, the MRL-Fas^lprcg ^mice exhibit sex differences in disease severity [37]. These observations suggest that reverse signaling through FasL is involved in generating sex differences in IgG isotypes, and consequently in the frequency of severe disease in female mice.

It has been shown that FasL expression in ovaries is closely correlated with estrogen levels, which vary at different phases of the female estrus cycle. This result suggests that estrogen dynamically controls FasL expression on various cells and may enhance Ig levels only once during each cycle [38]. To directly establish the role of estrogen in this reverse signaling pathway, we carried out *in vitro *stimulation of CD8^+ ^T cells and assessed Ig isotype levels. We have shown here that FasL expression on activated CD8^+ ^T cells is influenced by estrogen and have further demonstrated that the culture supernatants from estrogen-activated CD8^+ ^T cells produce growth factors that enhance *in vitro *immunoglobulin levels. These data suggest that reverse signaling through FasL in CD8^+ ^T cells leads to the production of growth factors that enhance the expression of Ig isotypes and that females are expected to have enhanced Ig switching because of their elevated post-pubertal estrogen levels. It is likely that some of the growth factors secreted by the activated CD8^+ ^T cells also influence B cell growth, maturation and differentiation.

In addition to their effects on CD8^+ ^T cells, estrogens affect the production of IFN-γ [39,40], which is known to enhance IgG2a responses [41]. These activated CD8^+ ^T cells would be expected to secrete growth factors and cytokines, which in turn would affect B cell growth and differentiation, leading to the enhanced immunoglobulin isotype expression in post-pubertal female mice. We therefore assessed the effect of IFN-γ on IgG2a levels in B6 IFN-γ knockout mice. These data suggested that increases in post-pubertal Ig isotype levels may be due to differential expression of cytokines (e.g., IFN-γ) produced by CD8^+ ^T cells activated through Fas-FasL reverse signaling. Recently, it has been shown that IgG2a-chromatin immune complexes, together with TLR 9 are very efficient in activating autoreactive B cells [42]. Our findings suggest that the increased IgG2a induced by the estrogen-Fas/FasL- IFN-γ pathway in post-pubertal female mice is one of the susceptibility factors enhancing autoimmunity in females. We speculate that differential expression of cytokines such as TGF-β may be involved in generating IgG2b differences in post-pubertal life.

Ig genes are transiently increased at the time of puberty in male mice (Figure 1A). The exact mechanism by which this increase occurs is not known. It is likely that the transiently elevated levels of estrogen at the time of puberty in males [43,44] may enhance FasL expression on CD8^+ ^T cells. Reverse signaling through FasL may also be responsible for this transient increase in Ig gene expression in male pubertal mice. The molecular basis for the large increase in innate immune response genes in males as compared to the adaptive immune response genes in females is not clear. It is possible that male hormones may regulate some of the innate immune response genes directly.

While the pathway analysis presented here has focused on the estrogen-Fas/FasL- IFN-γ pathway, our data also have implications with regard to male-related immunity. It has been observed that males have a higher mortality due to infectious diseases than do females [45], in part because of testosterone-induced immunosuppression in post-pubertal males [46]. The exact molecular mechanisms by which testosterone suppresses the acquired immune system are not yet understood. The data presented here suggest that males have an adequate innate immune response (first line of defense) but a relatively diminished adaptive immune response, which is critical for the elimination of the microorganisms. Thus, the documented higher mortality rates in males worldwide may be due in part to this relatively deficient adaptive immune response.

## Conclusion

We have shown that male and female mice differ significantly in post-pubertal life with respect to their immune functions. We have defined one key molecular pathway in this sexual dimorphism, in which we have attributed a novel function to the Fas-FasL pathway, enhancing immunoglobulin gene expression in post-pubertal female mice. These findings have clear implications not only for studies of autoimmunity but also for transplantation and vaccination.

## Methods

### Mice

Pre-pubertal (3- to 4 week-old), pubertal (6- to 9-week-old) and post-pubertal (16- to 20-week- and 24- to 28-week-old) C57BL/6 (B6) mice (The Jackson Laboratory) were used for gene expression profiling experiments. Johns Hopkins University is an AAALAC-accredited institution, and the mice were housed and cared for in accordance with institutional guidelines.

### Gene expression profiling and analysis

Expression profiling using Affymetrix U74Av2 (12,488 probe sets) was done as previously described [47]. In brief, six spleens from female and male B6 mice in each age group (pre-pubertal, pubertal and post-pubertal) (a total of 36 mice) were used for expression profiling. The spleens were homogenized in guanidinium thiocyanate homogenization buffer (0.1 M Tris HCl, pH 7.5, with 4.0 M guanidinium thiocyanate and 1% β-mercaptoethanol) using a Polytron homogenizer (Brinkmann). Total RNA was extracted by centrifuging the homogenate at 25,000 rpm for 24 h over a CsCl cushion (5.7 M CsCl with 0.01 M EDTA, pH 7.5). Double-stranded cDNA was synthesized from each aliquot using 8 ug of total RNA and the SuperScript Choice system (Invitrogen) and T7-(dT_24_) primer (GENESET Corp). Double-stranded cDNA reactions and all the following steps were done in duplicate for each sample. Double-stranded cDNA was purified using Phase Lock Gel (Eppendorf-5 Prime). Biotin-labeled cRNA was then synthesized from the double-stranded cDNA by *in vitro *transcription using a BioArray HighYield RNA Transcript Labelling Kit (Affymetrix). The cRNA was then purified using an RNeasy Mini kit (QIAGEN), fragmented, and hybridized to murine genome U74A chips for 16 h. The GeneChips were then washed and stained on the Affymetrix Fluidics Station 400 following Affymetrix protocols. The stained images were read using a Hewlett-Packard G2500A Gene Array Scanner and stored in an Affymetrix Microarray Laboratory Information Management System (LIMS). Quality control measures included >4-fold cRNA amplification (from total RNA/cDNA), scaling factors <2 to reach a whole-chip normalization of 800, and visual observation of hybridization patterns for chip defects. Probe set analysis was done using Microarray Suite version 5.0. The signal intensity values (absolute analyses) of the probe sets were then loaded into GeneSpring (Silicon Genetics, Redwood city, CA) for further analysis. Gene clusters were identified using statistical analysis of expression based on correlation coefficient. Briefly, a gene differentially regulated at a specific age was selected, and then a gene cluster was generated whose expression pattern correlates to the selected gene with the correlation coefficient of 0.97. All data files available through Public Expression Profiling Resource [48].

### Determination of serum polyclonal isotype-specific Ig levels

Serum samples from the various age groups (B6, B6 *lpr *and B6 *gld *mice [16–20 weeks old] and B6 GKO mice [16 weeks old]) were collected and stored in aliquots at -80°C before analysis. The levels of serum polyclonal IgG1, IgG2a, IgG2b, IgG3, IgM, IgA, kappa and lambda light chain antibodies were determined using isotype-specific antibodies. Ig levels in these sera were assayed by solid-phase enzyme-linked immunosorbent assay (ELISA) using goat anti-mouse Ig antibody-coated plates and alkaline phosphatase-conjugated isotype-specific anti-Ig antibodies as developing reagents (Southern Biotechnology). Dilutions of sera (IgG1, IgG2b and κ light chain at 1:10,000; 1:50,000 and 1:100,000; IgG2a, IgG3, IgM, IgA and λ light chain at 1:1,000, 1:5,000 and 1:10,000) from experimental mice were prepared, and the results are expressed as OD_405 _absorbance values.

### Determination of serum amyloid A and serum haptoglobin levels

Mouse serum amyloid A levels were determined using a solid-phase ELISA (Phage Range, Tridelta), and serum haptoglobin levels were determined using a colorimetric assay according to the manufacturer's instructions (Phage Range, Tridelta). Statistical significance was calculated using students t-test. A p value less than 0.05 was considered statistically significant.

### Flow cytometric analysis

All antibodies and reagents used for surface and intracellular cytofluorimetric analyses were purchased from Pharmingen. FasL expression was assessed on CD8^+ ^T cells after stimulation with combinations of anti-CD3/anti-CD28; FasFc; and estrogen using anti-FasL antibodies. Cell staining was detected by flow cytometry on FACS Calibur (Becton Dickinson) and analyzed using Cell Quest software.

### Purification of CD8^+ ^T cells and *in vitro *antibody synthesis assays

Spleens from post-pubertal (12- to 14-week-old) female mice were disrupted in PBS containing FBS. CD8^+ ^T cells were enriched using a Spin-Sep murine cell enrichment kit (Stem Cell Technologies) according to the manufacturer's protocol. T cells (5 × 10^5^) were stimulated for 18 h with various stimulants, either individually or in combinations (anti-CD3/anti-CD28; FasFc; and estrogen). These activated CD8^+ ^T cells were washed and added to 1.5 × 10^6 ^splenocytes in 24 well plates. On day 3 half of the medium was removed and supplemented with fresh medium and further incubated for 7 days. On day 10 the culture supernatants were collected and assayed for Ig isotypes.

### Determination of IFN-gamma in culture supernatants

Purified CD8^+ ^T cells were stimulated with plate-bound anti-CD3/anti-CD28 (2.5 μg/ml/10 μg/ml) and FasFc (2.5 and 1.25 μg/ml) in the presence and absence of estrogen (1 × 10^-8^M) for 24 h, and the supernatants were assayed for IFN-gamma using a commercial ELISA kit (Quantikine IFN-γ, R&D Systems). Statistical significance was calculated using students t-test. A p value less than 0.05 was considered statistically significant.

### *In vitro *TLR stimulation of splenocytes

Total splenocytes were isolated from post-pubertal male and female B6 mice as described above. Cells (2.5 × 10^6 ^cells/ml) were stimulated with the following doses of TLR ligands: lipopolysaccharide (LPS, Sigma), 200 ng/ml; lipoteichoic acid (LTA, Sigma), 5 μg/ml; Poly I:C (Sigma), 50 μg/ml; Pam3CSK4 (EMC Microcollections), 1 ng/ml; Imiquimod, 100 ng/ml; T1 CpG DNA (5'-TCGTCGTTTTGTCGTTTTGTCGTT-3'), 400 ng/ml. After two day incubation with these ligands, supernatants were isolated, and cytokine and chemokine analyses were carried out using SearchLight Technology (Pierce Biotechnology). This system uses multiplexed sandwich ELISAs to quantify up to 16 different cytokines/chemokines per well of a 96-well plate. The results were expressed as pg/ml (mean ± SD). Statistical significance was calculated using students t-test. A p value less than 0.05 was considered statistically significant.

## Authors' contributions

RL conducted research, analyzed data and wrote the paper. PZ, RR, AD, JCH, JJC, RA conducted research. PC analyzed data and critically evaluated the paper. AR, EPH designed research and critically evaluated the paper. KN oversaw research, designed and conducted experiments, analyzed data, and wrote the paper.

## Supplementary Material

Additional File 1 Genes up regulated during puberty in male and female mice. Click here for file

Additional File 2 Genes down regulated during puberty in male and female mice. Click here for file

Additional File 3 Genes differentially expressed in post-pubertal male and female mice. Click here for file

Additional File 4 **Effect of estrogen on FasL expression in activated CD8^+ ^T cells**. Purified CD8^+ ^T cells were isolated and stimulated with plate-bound CD3/CD28 in the presence and absence of estrogen (10^-8^M) for 24 h. The cells were stained with PE-labeled anti-FasL antibodies. Filled area, isotype control; green line, CD3/CD28-stimulated cells; pink line, CD3/CD28 and estrogen. Click here for file

Additional File 5 **IFN-γ levels in activated CD8^+ ^T cells**. Purified CD8^+ ^T cells were cultured in the presence and absence of plate-bound Fas-Fc/CD3/CD28 in the presence and absence of estrogen. IFN-γ was estimated using a commercial ELISA kit. Click here for file
