# Supplementary material for: Sexual dimorphism in immune response genes as a function of puberty
Source: BMC Immunol. 2006 Feb 22;7:2. doi: 10.1186/1471-2172-7-2 (PMC1402325; doi:10.1186/1471-2172-7-2)
Supplement: Additional File 1 — Genes up regulated during puberty in male and female mice. [file 1471-2172-7-2-S1.doc]

Genes up regulated during puberty in male and female mice.

| Acc # | **Male Fold Change** | **Female Fold Change** | **Gene Name** |
| --- | --- | --- | --- |
| AB023622 | 1.9 | 2.3 | Septin6 |
| AF002701 | 3.1 | 10.6 | Glial cell line derived neurotrophic factor family receptor alpha 2 |
| U65592 | 3.4 | 1.4 | K+ channel beta2 subunit |
| M21019 | 1.9 | 1.8 | Harvey rat sarcoma oncogene |
| L03547 | 3.6 | 2.0 | Early lymphoid specific transcription factor |
| D25540 | 4.5 | 6.6 | TGF-beta type I receptor |
| M16118 | 2.1 | 1.7 | T-cell receptor insulin (A-chain) reactive alpha chain VJC |
| J00475 | 2.6 | 1.5 | Mouse germline IgH chain gene, DJC region- segment D-FL16.1 |
| U12919 | 1.7 | 1.4 | Adenylate cyclase 7 |
| X73230 | 2.7 | 2.0 | Arylsulfatase A |
| M58482 | 1.4 | 1.7 | Mouse avian erythroblastosis virus E26 oncogene homolog 1 (ets-1) |
| L26528 | 2.3 | 1.7 | RAB11B, member RAS oncogene family |
| AF042158 | 1.4 | 1.5 | MHC class II transactivator CIITA form III |
| X59990 | 3.6 | 1.8 | Catenin alpha 1 |
| J00475 | 2.6 | 1.5 | Mouse germline IgH chain gene, DJC region- segment D-FL16.1 |
| U18424 | 2.4 | 1.8 | Bacteria binding macrophage receptor MARCO |
| D89866 | 2.0 | 2.5 | Ceramide glucosyltransferase |
| D17571 | 2.0 | 1.7 | P450 (cytochrome) oxidoreductase |
| X80903 | 1.4 | 2.0 | Delta-like 1 homolog (Drosophila) |
| U40720 | 2.5 | 2.1 | Homeo box gene expressed in ES cells |
