# Supplementary material for: Sexual dimorphism in immune response genes as a function of puberty
Source: BMC Immunol. 2006 Feb 22;7:2. doi: 10.1186/1471-2172-7-2 (PMC1402325; doi:10.1186/1471-2172-7-2)
Supplement: Additional File 2 — Genes down regulated during puberty in male and female mice. [file 1471-2172-7-2-S2.doc]

Genes down regulated during puberty in male and female mice.

| **Acc. #** | Male Fold Change | **Female Fold Change** | **Gene Name** |
| --- | --- | --- | --- |
| X92410 | -2.5 | -1.4 | RAD23a homolog |
| X82786 | -3.6 | -3.2 | Ki 67 |
| AJ009840 | -7.0 | -3.5 | Cathepsin E gene |
| Y15003 | -3.2 | -2.7 | Beta-galactoside alpha-2,3-sialyltransferase |
| U88588 | -4.8 | -4.2 | Cerebellar degeneration-related 2 |
| X53081 | -2.3 | -1.7 | Erythropoietin receptor |
| AE000665 | -1.4 | -2.1 | TCR beta locus |
| U17297 | -1.5 | -1.5 | Erythrocyte protein band 7.2 |
| M25944 | -3.4 | -2.4 | Carbonic anhydrase II (CAII) |
| M61215 | -2.3 | -2.1 | Ferrochelatase |
| U85414 | -1.7 | -1.8 | Glutamate cysteine ligase |
| M15268 | -3.3 | -3.8 | Aminolevulinic acid synthase 2, erythroid |
| AF094519 | -3.3 | -2.1 | Diaphanous-related formin (Dia2) |
| L02914 | -5.2 | -3.5 | Aquaporin 1 |
| X02677 | -4.6 | -5.0 | Solute carrier family 4 (anion exchanger) |
| U12961 | -3.1 | -2.2 | NAD(P)H menadione oxidoreductase 1 |
| AF057524 | -6.8 | -4.4 | Membrane protein Rh30 (Rhl) |
| AJ006215 | -2.9 | -2.2 | CMP-N-acetylneuraminic acid synthetase |
| U53586 | -1.9 | -1.8 | Ecotropic viral integration site 5 |
| D16333 | -2.0 | -1.7 | Coproporphyrinogen oxidase |
| AB008674 | -1.4 | -2.0 | mBlm |
| AB025411 | -2.1 | -1.7 | Ten-m2 |
| U19604 | -1.6 | -1.5 | DNA ligase I, ATP-dependent |
| AF029347 | -2.2 | -1.9 | Chloride channel protein 3 (CLCN3) |
| X83577 | -1.8 | -1.6 | Glypican 4 |
| X13586 | -3.4 | -3.6 | 2,3-bisphosphoglycerate mutase |
| U89155 | -2.9 | -2.7 | Untitled |
| U95607 | -3.8 | -2.3 | Testis specific DNAj-homolog |
| D16333 | -2.0 | -1.7 | Coproporphyrinogen oxidase |
| U42385 | -2.2 | -2.1 | Fibroblast growth factor inducible 16 |
| U01915 | -2.3 | -1.7 | Topoisomerase (DNA) II alpha |
| U62021 | -1.9 | -1.6 | Euronal pentraxin 1 |
| D49733 | -2.3 | -1.9 | Lamin A |
| U51866 | -1.6 | -1.5 | Casein kinase II alpha subunit |
| X66032 | -2.7 | -2.1 | Cyclin B2 |
| L35049 | -2.5 | -2.7 | Bcl2-like |
| X13752 | -3.6 | -3.2 | Delta-aminolevulinate dehydratase |
| AJ133430 | -1.6 | -1.4 | Or6 gene |
| U34691 | -5.2 | -4.2 | Uroporphyrinogen decarboxylase |
| Z35294 | -2.3 | -1.6 | MTCP-1 gene |
| AJ223087 | -1.9 | -2.1 | C58-related protein |
| U32446 | -2.4 | -1.8 | Breast cancer 1 |
| AF002823 | -1.6 | -1.9 | Budding inhibited by benzimidazoles homolog |
| V00716 | -1.8 | -1.8 | Pseudogene for alpha-globin |
| AF032714 | -3.5 | -2.0 | Type II peroxiredoxin protein 2 (PrxII-2) pseudogene |
| L29480 | -2.0 | -2.2 | Serine/threonine kinase (sak-b) |
| M28666 | -6.5 | -3.5 | Hydroxymethylbilane synthase |
| AB008895 | -1.6 | -2.0 | mGpi1p |
| U07861 | -1.4 | -1.5 | Zinc finger protein 101 |
| M26385 | -6.5 | -5.1 | Glycophorin A |
| D12646 | -1.5 | -2.6 | Kinesin heavy chain member 4 |
| AF017275 | -2.4 | -2.1 | Growth factor independent 1B |
| D13695 | -3.1 | -1.9 | Lymphocyte antigen 84 |
| AF032128 | -2.2 | -2.1 | Antizyme inhibitor |
| AF103875 | -2.9 | -2.2 | Placenta-specific ATP binding cassette transporter |
| D87902 | -1.6 | -1.4 | ARF5 |
| M38724 | -3.1 | -2.2 | Cell division cycle control protein 2a |
