# Supplementary material for: Sexual dimorphism in immune response genes as a function of puberty
Source: BMC Immunol. 2006 Feb 22;7:2. doi: 10.1186/1471-2172-7-2 (PMC1402325; doi:10.1186/1471-2172-7-2)
Supplement: Additional File 3 — Genes differentially expressed in post-pubertal male and female mice. [file 1471-2172-7-2-S3.doc]

Genes differentially expressed in post-pubertal male and female mice.

| **Acc #** | **Male Fold Change** | **Female Fold Change** | Gene name |
| --- | --- | --- | --- |
| X03505 | 95.9 | 8.7 | Serum amyloid A |
| M96827 | 3.6 | 0.7 | Haptoglobin |
| AF076482 | 3.6 | 0.7 | Peptidoglycan recognition protein |
| AF071180 | 3.0 | 1.4 | Formyl peptide receptor-1like receptor |
| X70920 | 2.7 | 0.6 | Granulocyte maturation Ly-6G.1 gene |
| M69260 | 3.5 | 1.0 | Lipocortin I gene |
| U49513 | 4.8 | 1.4 | MIP-1gamma |
| X81627 | 3.8 | 0.5 | 24p3 gene |
| U29678 | 5.7 | 1.9 | Mip1-alpha/Rantes receptor; CCR-1 |
| AA144469 | 4.1 | 0.7 | Interferon-inducible protein 1-8U |
| AA596710 | 4.8 | 0.9 | Leukotriene B4 12-hydroxydehydrogenase |
| X59769 | 3.4 | 1.3 | IL-1r2 mRNA for type II interleukin-1R |
| U05265 | 3.2 | 1.9 | gp49B gene |
| AF051367 | 1.7 | 1.1 | Integrin beta subunit-like cell-surface protein |
| AF061972 | 1.8 | 0.9 | CC3/TIP30 |
| AF084575 | 2.5 | 1.0 | AP-3 heterotetrameric complex |
| J03298 | 1.7 | 0.5 | Lactotransferrin precursor |
| M65027 | 3.4 | 2.4 | Cell surface antigen gp49 mRNA |
| M27008 | 10.3 | 0.9 | Alpha-1 acid glycoprotein (Agp-1B) |
| AF099977 | 2.3 | 1.6 | mSLFN4 |
| J05018 | 6.6 | 1.7 | High affinity IgE receptor alpha subunit |
| D63357 | 3.1 | 2.2 | RegIIIalpha protein |
| AA871791 | 2.5 | 1.3 | Barstead bowel MPLRB9 |
| X16490 | 2.7 | 1.0 | Plasminogen activator inhibitor 2 |
| AF004428 | 2.4 | 1.9 | D53; TPD52L1 |
| AF078752 | 2.1 | 1.2 | DGAT |
| X066449 | 2.1 | 1.2 | Calcyclin |
| AF069708 | 6.2 | 1.6 | Stathmin family of proteins |
| AU371861 | 6.1 | 2.5 | RegIIIbeta/PAP protein |
| J05018 | 6.6 | 1.7 | High affinity IgE receptor alpha |
| X93035 | 4.6 | 0.4 | Glycosyl hydrolases |
| X15592 | 2.5 | 1.8 | Ctla-2-beta |
| U96696 | 18.9 | 0.5 | neutrophil collagenase (MMP 8) |
| X70057 | 12.9 | 0.9 | Cathepsin G |
| U04962 | 5.4 | 0.7 | neutrophil elastase |
| X15313 | 4.4 | 0.6 | myeloperoxidase |
| U43525 | 6.7 | 0.7 | protease 3 |
| AE000665 | 1.1 | 2.4 | TCR beta locus |
| X00651 | 1.6 | 4.0 | Ig-kappa light chain V-J kappa 5 joining region |
| AB017349 | 1.5 | 3.1 | mRNA for immunoglobulin light chain V region |
| U19315 | 1.9 | 4.2 | Immunoglobulin kappa light chain variable region |
| AF045024 | 1.7 | 4.7 | Immunoglobulin kappa light chain variable |
| M15593 | 2.0 | 4.9 | Ig kappa chain 7B6 mRNA |
| AF045026 | 1.8 | 4.5 | Immunoglobulin kappa light chain variable |
| AE000664 | 0.9 | 2.0 | TCR beta locus from bases |
| X88903 | 1.9 | 6.3 | Variable light chain (1.7) |
| X16678 | 1.5 | 2.9 | Immunoglobulin kappa chain variable 20 |
| X00652 | 1.5 | 3.0 | Ig-kappa light chain V-J kappa 5 joining region |
| M90766 | 1.6 | 3.3 | Ig active joining chain mRNA of the b allele |
| AF037206 | 0.7 | 1.7 | RING zinc finger protein (Rzf) |
| M86751 | 1.4 | 2.1 | Ig L-chain gene variable region |
| L28095 | 1.3 | 2.3 | Interleukin 1-beta converting enzyme |
| X94420 | 1.4 | 4.7 | IgA V-D-J-heavy chain |
| V00793 | 56.1 | 141.8 | IgG1 |
| X67210 | 5.9 | 13.3 | IgG2 |
| L43568 | 1.3 | 5.3 | B-cell receptor gene |
| J00389 | 1.4 | 2.1 | Glandular kallikrein |
| M13500 | 1.1 | 2.0 | Potential kallikrein gene |
| U37386 | 0.5 | 3.0 | Carboxyl ester lipase |
| D85605 | 1.3 | 3.7 | Cholecystokinin A receptor |
| U28419 | 0.9 | 3.0 | Eukaryotic translation initiation factor 1A |
| X51942 | 0.9 | 1.5 | Phenylalanine hydroxylase |
| M55412 | 0.7 | 1.6 | Guanine nucleotide binding protein, |
| U37438 | 1.6 | 2.6 | CRP-ductin-alpha mRNA |
| X04725 | 0.8 | 4.2 | Insulin I |
| X51547 | 1.4 | 3.0 | Lzp-s mRNA for lysozyme P |
| M17979 | 1.2 | 2.2 | Epidermal growth factor binding protein type A |
| Y00500 | 1.2 | 2.7 | Kallikrein 5 |
| D14011 | 2.0 | 3.3 | Rat regenerating islet-derived, mouse homolog 2 |
| M13500 | 1.1 | 2.0 | Potential kallikrein gene |
| V00829 | 1.2 | 2.5 | mGK-1 (complete gene) and mGK-2 fragment |
| M17962 | 1.4 | 4.4 | Epidermal growth factor binding protein type C |
| X78545 | 1.9 | 1.1 | Mast cell protease 8 |
| M94584 | 6.7 | 0.4 | Chitinase 3-like 3 |
