# Supplementary figures and images for: Sexual dimorphism in immune response genes as a function of puberty
Source: BMC Immunol. 2006 Feb 22;7:2. doi: 10.1186/1471-2172-7-2 (PMC1402325; doi:10.1186/1471-2172-7-2)

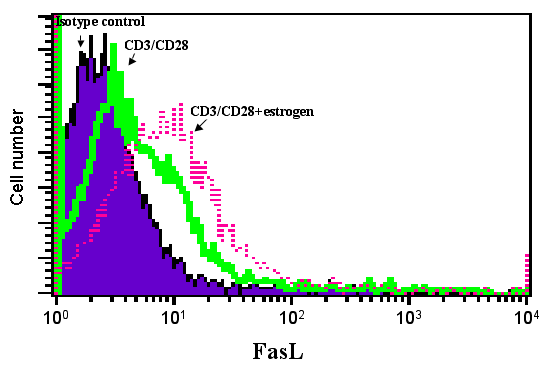

Supplement: Additional File 4 — Effect of estrogen on FasL expression in activated CD8+ T cells. Purified CD8+ T cells were isolated and stimulated with plate-bound CD3/CD28 in the presence and absence of estrogen (10-8M) for 24 h. The cells were stained with PE-labeled anti-FasL antibodies. Filled area, isotype control; green line, CD3/CD28-stimulated cells; pink line, CD3/CD28 and estrogen. [file 1471-2172-7-2-S4.tiff]

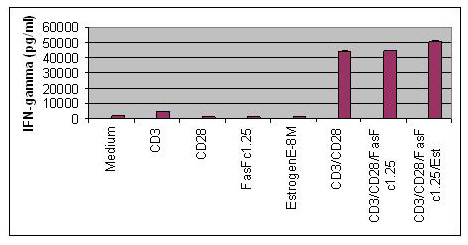

Supplement: Additional File 5 — IFN-γ levels in activated CD8+ T cells. Purified CD8+ T cells were cultured in the presence and absence of plate-bound Fas-Fc/CD3/CD28 in the presence and absence of estrogen. IFN-γ was estimated using a commercial ELISA kit. [file 1471-2172-7-2-S5.jpeg]
